# Supplementary material for: RAD51D Aberrant Splicing in Breast Cancer: Identification of Splicing Regulatory Elements and Minigene-Based Evaluation of 53 DNA Variants
Source: Cancers (Basel). 2021 Jun 7;13(11):2845. doi: 10.3390/cancers13112845 (PMC8201001; doi:10.3390/cancers13112845)
Supplement: Supplementary file 1 [file cancers-13-02845-s001.zip › cancers-1205645-supplementary.pdf]

Supplementary Materials: RAD51D Aberrant Splicing in Breast Cancer: Identification of Splicing Regulatory Elements and Minigene-Based Evaluation of 53 DNA Variants

Elena Bueno-Martínez, Lara Sanoguera-Miralles, Alberto Valenzuela-Palomo, Víctor Lorca, Alicia Gómez-Sanz, Sara Carvalho, Jamie Allen, Mar Infante, Pedro Pérez-Segura, Conxi Lázaro, Douglas F. Easton, Peter Devilee, Maaike P. G. Vreeswijk, Miguel de la Hoya and Eladio A. Velasco

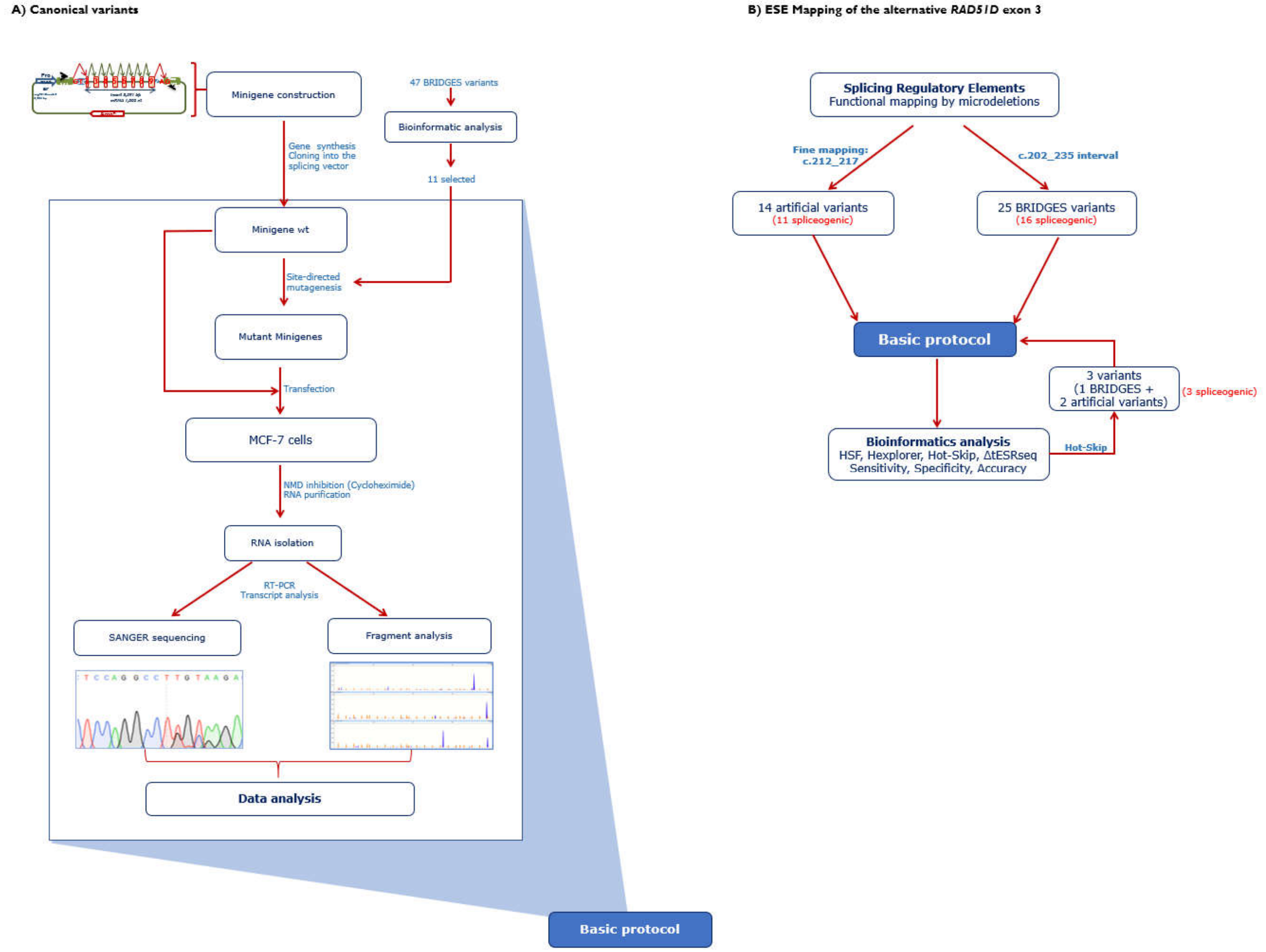

Figure S1. Workflow of the minigene protocol. (**A**) Basic protocol for the analysis of canonical BRIDGES variants. The basic assay includes the following steps: (1) Minigene construction; (2) Site-directed mutagenesis; (3) Transfection of the wild type and mutant minigenes; (4) Inhibition of Nonsense-mediated decay and RNA purification; (5) Transcript sequencing and fragment analysis by fluorescent capillary electrophoresis; (6) Data interpretation. (**B**) Analysis of Splicing Regulatory Elements. Functional mapping of exon 3 by microdeletions was performed and the interval c.202_235 showed the greatest splicing impact without any trace of the full-length transcript. Then, 25 non-canonical BRIDGES exon 3 variants from c.202_235 and surrounding sequences were tested. In addition, a fine mapping of the c.202_235 interval with additional 10–11 bp microdeletions was performed so that we proceeded to test all the possible single-nucleotide substitutions between c.212_217 within the highest spliceogenic interval. These total 18 possible variants, four of which were in the pool of the 25 BRIDGES variants previously tested, resulting in 14 artificial (non-reported) variants to be evaluated. To check the sensitivity, specificity and accuracy of splicing enhancers/silencers predictors, a bioinformatics analysis was carried out with HSF, Hexplorer, Hot-Skip, ΔtESRseq. As a result of this study, Hot-skip suggested 3 changes (1 reported

in BRIDGES) with the highest probabilities to disrupt splicing, so they were also assayed. The number of spliceogenic variants are shown in red between parentheses.

```
SacII
CCGCGGtcctagagggccgcgcttctggagaggctcaggaagggtgggcgtggggagccaagctctaaaacag
ggtagagattcctactgtgtgcagggcaccgcgccggctcctgcagctttgttatttcacactggcctcctga
gacggcctgccgggggtagaattgacaccccatttgacagatgaggaaaaaccaagtgtgggaggccccagt
cctctctgccaatccgctatacgtgttttgttttcagTGGTGGACCTGGTTTCTGCAGACCTGGAAGAGGTAG   Ex2
CTCAGAAATGTGGCTTGTCTTACAAGgtgagctcctgatctccacattccaaacctgagtgtctttttaaaaa
gtccatcccaagccagctgggcgcggtggctcatgcctgtaatcccagcactttgggaggccgaggcgggtgg
gtcacttggagtcagaagtcaggagttcgagaccatccaggccaacatggtgaaaccctgtccctactggcag
aggttgcagtgagccgagatcgtgctactgcactccaatctgggcgacagagtgagaccctgtctgaaacaac
aacaataacaaaagtccatcccaagccaaagggagcagagggttctcagatattgagaagtagggtgaatgac
accctgggatgccagcttctacgggcaatttgttgctgagcagtctggggccctttggcagGCCCTGGTTGCC
CTGAGGCGGGTGCTGCTGGCTCAGTTCTCGGCTTTCCCCGTGAATGGCGCTGATCTCTACGAGGAACTGAAGA   Ex3
CCTCCACTGCCATCCTGTCCACTGGCATTGGCAGgtttgtgtgtgtacttggggaagaaggagagaggcagga
ggatggggcctcctctcagctctgcttttgatgcttgagtctagacgggaggggtttttttttaaccaataa
tggatgggaaggaaaggggtcaggacatctgaattcttatcctataacagggttatggggtatctcagccttg
gcactattgatatttgagggatgaagagagaccagagaaggcacatttgggcattccctccttaccagaagaa
aacaccaggagaagcctcctggccagtgatgttcaaagacagaaccagtgcttgaaagaaatgtcttacttttt
cttcttggtaccttttcccctgtcttcctcctaacctcttgtgttgatttcttctcttctgccatcagTCTT
GATAAACTGCTTGATGCTGGTCTCTATACTGGAGAAGTGACTGAAATTGTAGGAGGCCCAGGTAGCGGCAAAA   Ex4
CTCAGgtacatgtgaggccagcagtcaggaggatttaggggtgagggtggtagatgcatagcccagggtccct
gggggagcttcagcgtactaatgggttcagagcccaacagagagaagatgggtaataaggaatggggtcggga
ctcagcccatttgtgttgctggaatctgggcaaggtttggtggggagctcccatctggaccctctctgaagtg
tcaagttcatccaatgcccccacccccagGTATGTCTCTGTATGGCAGCAAATGTGGCCCATGGCCTGCAGCA
AAACGTCCTATATGTAGATTCCAATGGAGGGCTGACAGCTTCCCGCCTCCTCCAGCTGCTTCAGGCTAAAACC   Ex5
CAGGATGAGGAGGAACAGgtaagggcaggatgctgggttccattgagattctgacacaggaaaaccccattat
cccttaacattgtaagaatttgggttgaatagtcattccttgccttcaaacttgctgttctattaatacatct
cgtattccttaagccacccctgttgatgccatcatcccatttgggatgctactcacactgattcttccaggac
ctctttcctgctgagttctgagtactttacagctcagcacccaggcagccaccactgtgacaactgaccatta
gacaaagctcatttgccaccctggacctgagtccttgcatccaggtgcctcttccttctcagccttaccttga
ccccccctactccctcttatccaaagtccttgcttttcttttccaccgcttcagGCAGAAGCTCTCCGGAGGAT
CCAGGTGGTGCATGCATTTGACATCTTCCAGATGCTGGATGTGCTGCAGGAGCTCCGAGGCACTGTGGCCCAG   Ex6
CAGgtgagcctgctgttctgcccctccttgcttaattcagctttctaggtgctatctctgtgggcaggtgtcc
tactacagactgtttggaaatgcagatgtgcaatctggtgactccaactatgcttgctactcccaggatggaa
ttcccgagcacctaaatcacatacctcattgtttacatgttcatcttccctgctgaagtcttcattgaagtct
gttacacatgccaatgggacgagggagacattgctgctgatgacattctgtgatgcccgtgcagcttgctgta
tttgggatgggggggctatttcttactagttttagatagaaaagcaagcttgtcctgtaatgtgtcctagagg
ctgacaggttcatgagtgcttcccccacattccttcagGTGACTGGTTCTTCAGGAACTGTGAAGGTGGTGGT   Ex7
TGTGGACTCGGTCACTGCGGTGGTTTCCCCACTTCTGGGAGGTCAGCAGAGGGAAGgtgagtgatgtggcaga
gcctggggccagtggaatgtgacctcgcctcccagctctggagtctggtctctggccagccccatccctggtt
ccccactgccctccctctctgcttctcctcctcctgcagGCTTGGCCTTGATGATGCAGCTGGCCCGAGAGCT   Ex8
GAAGACCCTGGCCCGGGACCTTGGCATGGCAGTGGTGgtgaggaagcaggcttggccaatgaacttgatttct
agttattgcgagctctttatttcccttaaatgtcagcttctgaaccccaaagtggccctttaggaaagggaca
tatcccagcttccctgggtcagctacagctttgcaccataaatagctaagggaatcaggaatcgtcttgttct
ggttcgggtcaaacctgaggcatcgagatttagtggaaagagcggggcacaagtcaggaggcccaggttctag
ttccagacctgccattaggttttgtaaccttggggaagttatttctctgtgctaggcctctgttttcctctcc
gtaaaatgaagcggtagtaatcgatgtcctctatactagcattatggatctgtaagtctgtttctgctgcagG
TGACCAACCACATAACTCGAGACAGGGACAGCGGGAGGCTCAAACCTGCCCTCGGACGCTCCTGGAGCTTTGT   Ex9
GCCCAGCACTCGGATTCTCCTGGACACCATCGAGGGAGCAGGAGCATCAGGCGGCCGGCGCATGGCGTGTCTG
GCCAAATCTTCCCGACAGgtgagccagccccaggaagatgccagtaatcttgggccctggagggtggtttcca
tacccacagatgtcttcgagaagaactctttagaggctgaaaccttgcaactaaagaagctacccacttgggt
tgcttaactccactgactccGTCGAC
                    SalI
```

Figure S2. Insert sequence of minigene mgR51D_ex2–9. Exons 2 to 9 are indicated in upper case and cloning sites (SacII / SalI) are underlined. Structure of the insert (3603 bp): BamHI-ivs1 (200 bp)—ex2 (62 bp)—ivs2-1 (200 bp)//ivs2-2 (200 bp)—ex3 (119 bp)—ivs3-1 (200 bp)//ivs3-2 (200 bp)—ex4 (82 bp)—ivs4 (243 bp)—ex5 (135 bp)—ivs 5-1 (200 bp)//ivs5-2 (200 bp)—ex6 (96 bp)—ivs6-1 (200 bp)//ivs6-2 (200 bp)—ex7 (91 bp)—ivs7 (129 bp)—ex8 (71 bp)—ivs8-1 (200 bp)//ivs8-2 (200 bp)—ex9 (165 bp)—ivs9 (148 bp)—SalI (shortened introns are indicated by a double slash //).

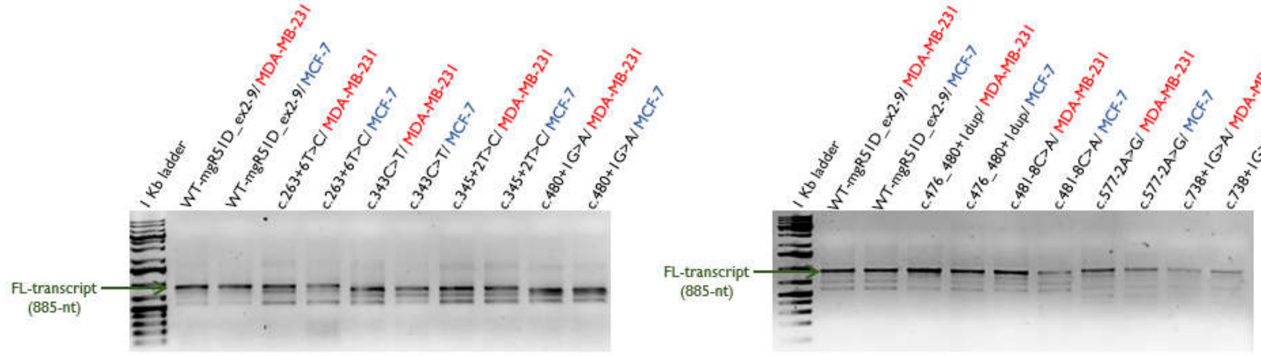

Figure S3. Agarose gel (1.0%) electrophoresis of the splicing assays of eight splice-site variants in MDA-MB-231 and MCF-7 cells. Cell growth and transfections were conducted as described in Section 2. Materials and Methods. The Gene Ruler 1 Kb Plus DNA Ladder (Thermo Scientific, Waltham, MA, United States) was employed as DNA size standard. RT-PCRs were carried out with primers RTR51D_ex2-fw and RTpSAD-RV (885 nt).

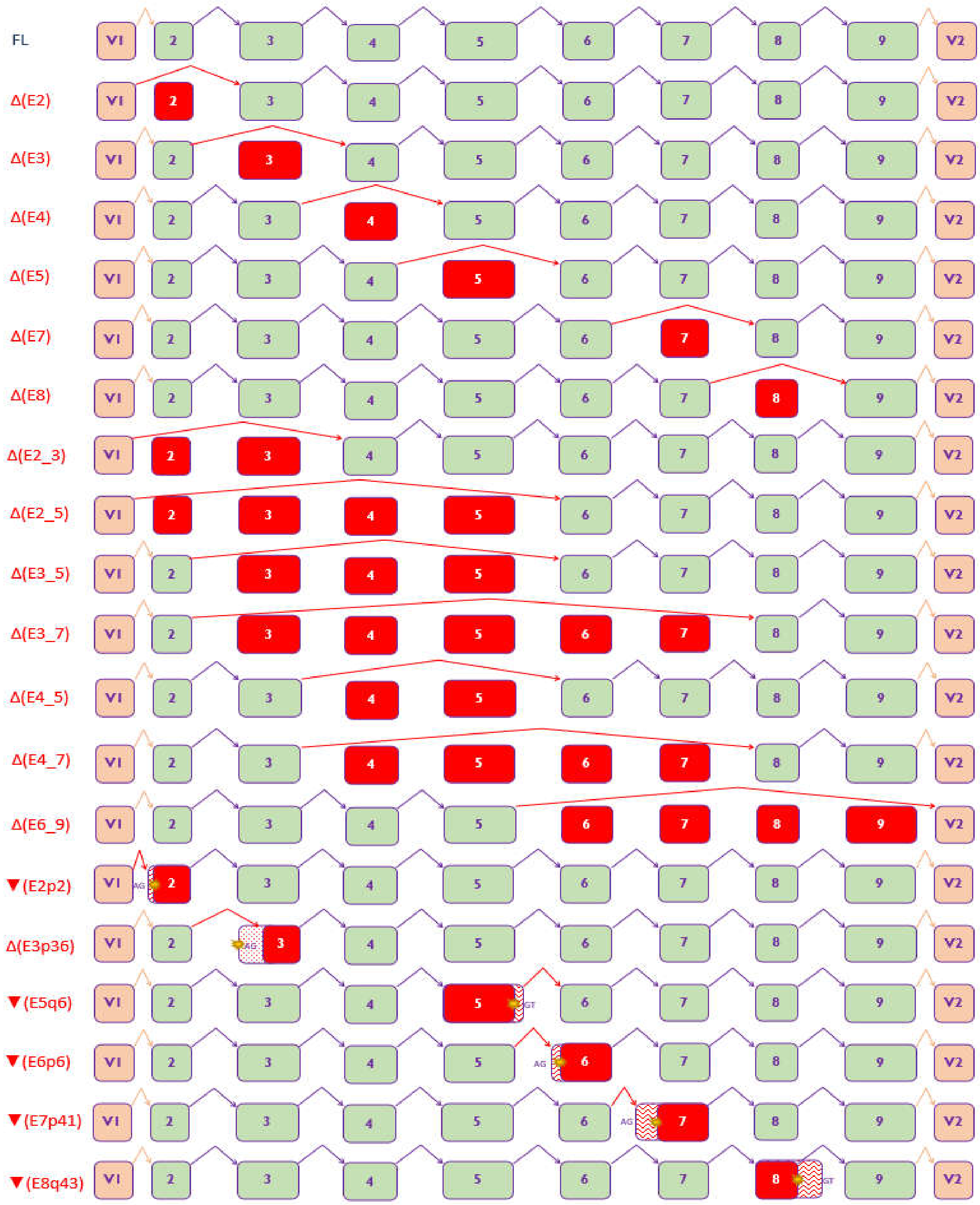

Figure S4. Schematic representation of the most common aberrant transcripts. Anomalous events are denoted by red-boxed exons and red-broken lines.

A

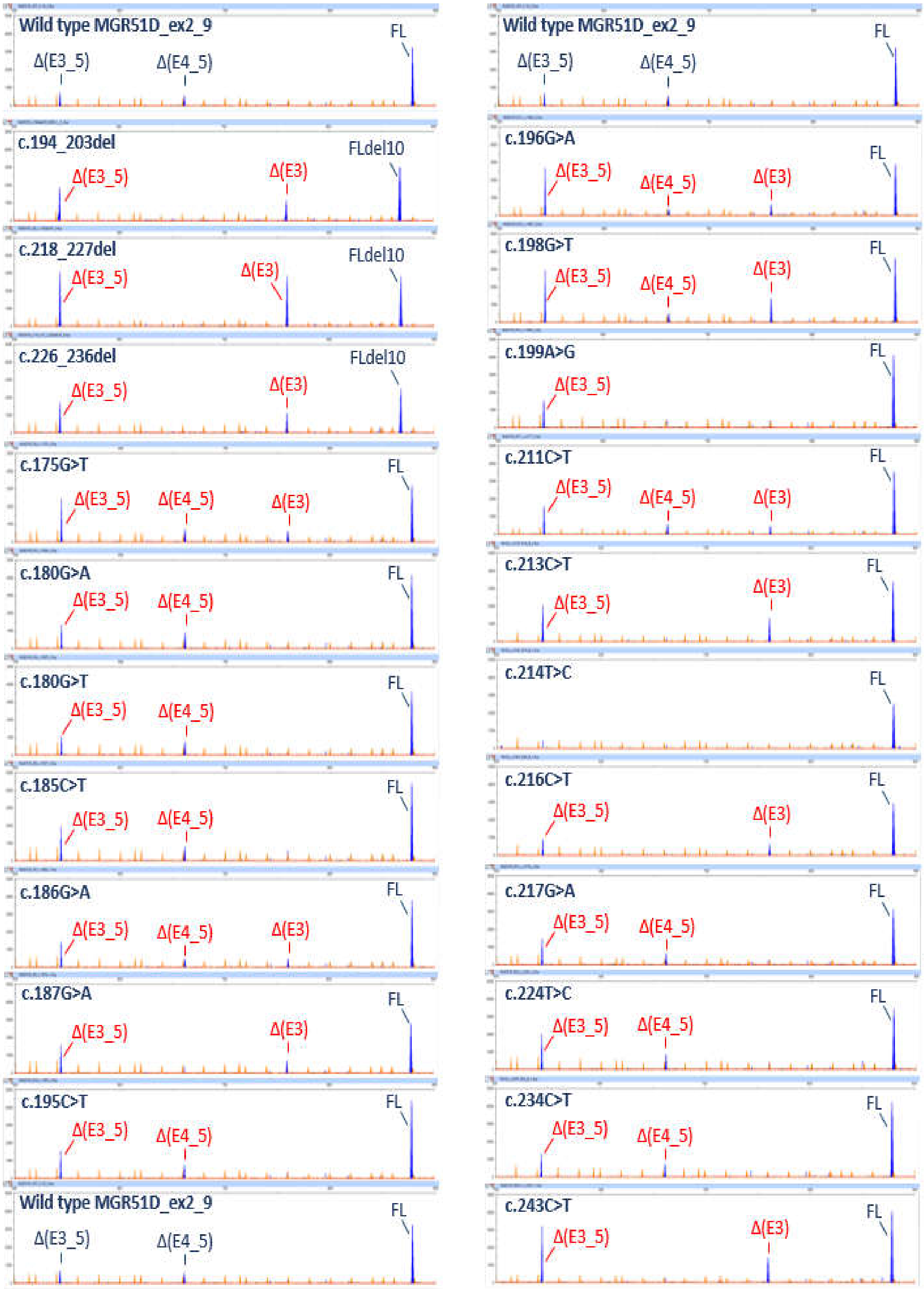
Wild type MGR51D_ex2_9
Δ(E3_5)
Δ(E4_5)
FL
c.194_203del
Δ(E3_5)
Δ(E3)
FLdel10
c.218_227del
Δ(E3_5)
Δ(E3)
FLdel10
c.226_236del
Δ(E3_5)
Δ(E3)
FLdel10
c.175G>T
Δ(E3_5)
Δ(E4_5)
Δ(E3)
FL
c.180G>A
Δ(E3_5)
Δ(E4_5)
FL
c.180G>T
Δ(E3_5)
Δ(E4_5)
FL
c.185C>T
Δ(E3_5)
Δ(E4_5)
FL
c.186G>A
Δ(E3_5)
Δ(E4_5)
Δ(E3)
FL
c.187G>A
Δ(E3_5)
Δ(E3)
FL
c.195C>T
Δ(E3_5)
Δ(E4_5)
FL
Wild type MGR51D_ex2_9
Δ(E3_5)
Δ(E4_5)
FL
Wild type MGR51D_ex2_9
Δ(E3_5)
Δ(E4_5)
FL
c.196G>A
Δ(E3_5)
Δ(E4_5)
Δ(E3)
FL
c.198G>T
Δ(E3_5)
Δ(E4_5)
Δ(E3)
FL
c.199A>G
Δ(E3_5)
FL
c.211C>T
Δ(E3_5)
Δ(E4_5)
Δ(E3)
FL
c.213C>T
Δ(E3_5)
Δ(E3)
FL
c.214T>C
FL
c.216C>T
Δ(E3_5)
Δ(E3)
FL
c.217G>A
Δ(E3_5)
Δ(E4_5)
FL
c.224T>C
Δ(E3_5)
Δ(E4_5)
FL
c.234C>T
Δ(E3_5)
Δ(E4_5)
FL
c.243C>T
Δ(E3_5)
Δ(E3)
FL

B

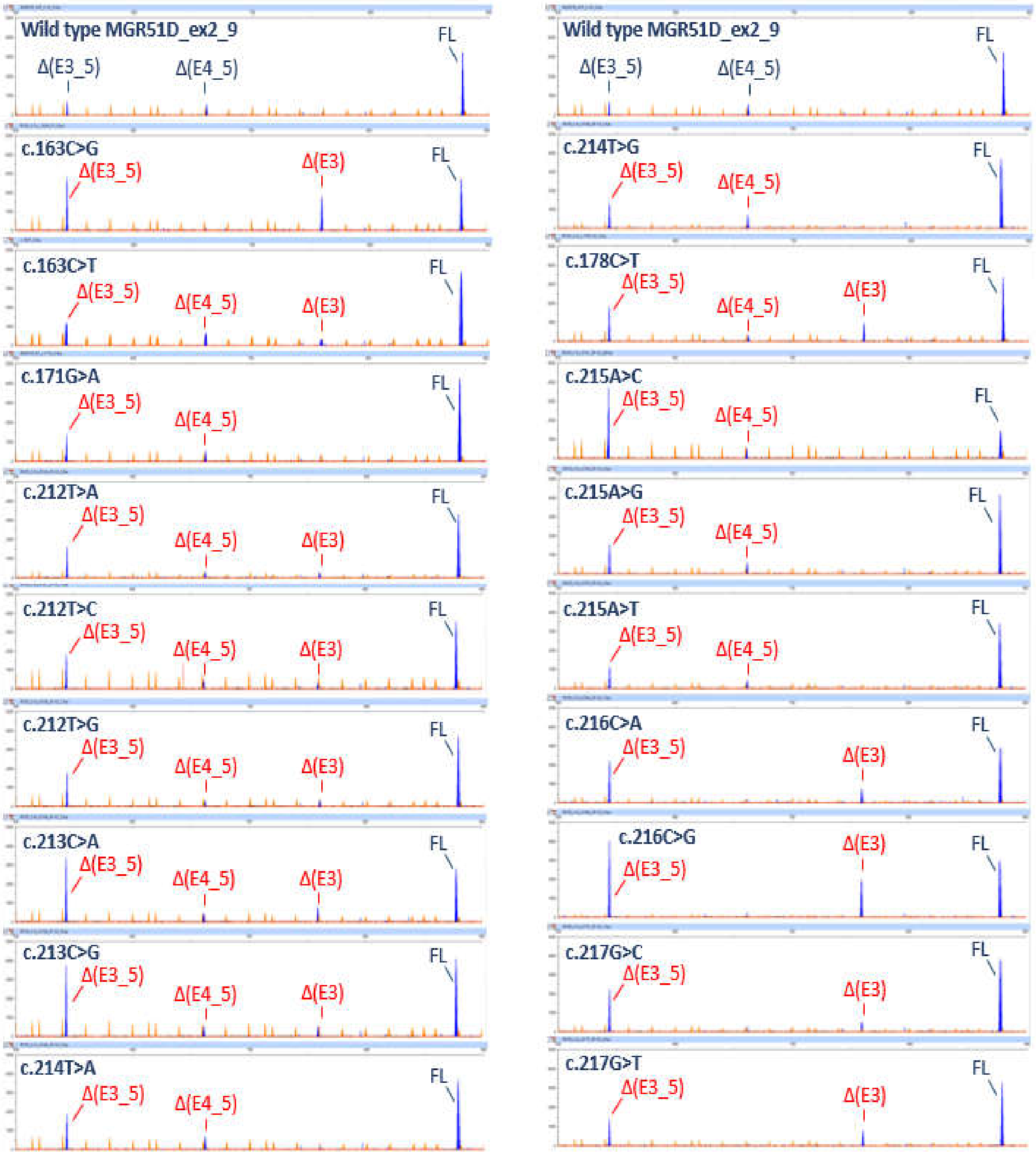

Figure S5. Fluorescent fragment analysis of other exon 3 microdeletions and variants. (**A**) Fluorescent fragment analysis of transcripts generated by selected microdeletion and mutant minigenes. cDNAs were amplified with primers RTR51D_ex2-fw and RTpSAD-RV. FAM-labelled products (blue peaks) were run with LIZ1200 (orange peaks) as size standard. (**B**) Fluorescent fragment analysis of other exon 3 microdeletions and variants. Fluorescent fragment analysis of transcripts generated by selected microdeletion and mutant minigenes. cDNAs were amplified with primers RTR51D_ex2-fw and RTpSAD-RV. FAM-labelled products (blue peaks) were run with LIZ1200 (orange peaks) as size standard.

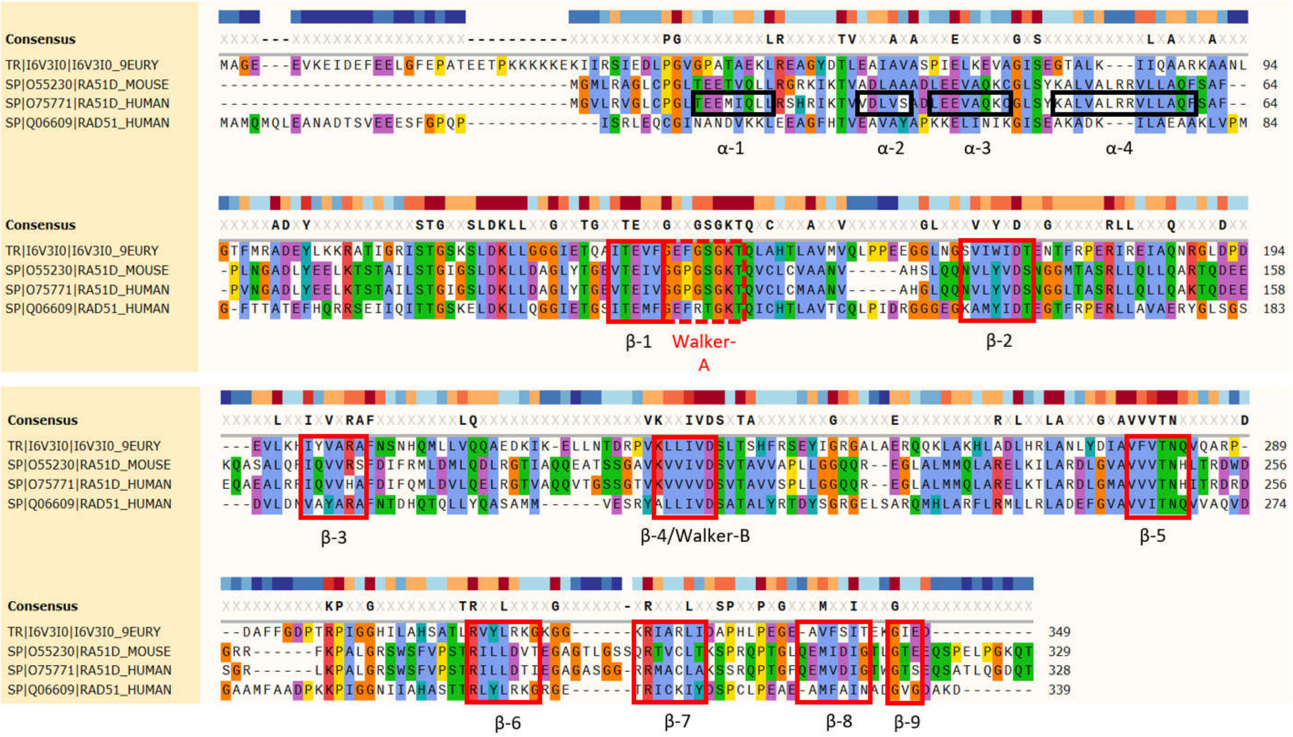

Figure S6. Mapping human RAD51D regions critical to protein function. Alignment of *P. furiosus* RAD51, Mus_musculus RAD51D, Homo_sapiens RAD51D, and Homo_sapiens RAD51. Left panel indicates the UNiProtKB identifier of the four aligned proteins. Alignment was performed online with the UniProt Align tool (Clustal Omega program; https://www.uniprot.org/align/, accessed on 1 April 2021). Alignment Visualization has been performed with SnapGene viewer. Colored bars indicate sequence conservation. Color code was set as properties + conservation (ClustalX). An NMR-based structural analysis of human RAD51D N-terminal domain has been reported Kim et al. [61]. According to this study, four α–helix (black boxes) are critical to support the structure and function of this ssDNA binding domain. By contrast, no structural analysis of human RAD51D C-terminal domain has been reported. Mapping of human RAD51D β–strands predicted critical to support C-terminal structure (red boxes), has been performed based on Mus_musculus Rad51d annotations previously reported by Miller et al. [62], and based on a *P. furiosus* Rad51 X-ray structure (Shin et al. [63]). Left panel indicates the UniProtKB identifier of the four aligned proteins. The role of the RAD51D Walker-A motif in HR, if any, is controversial.

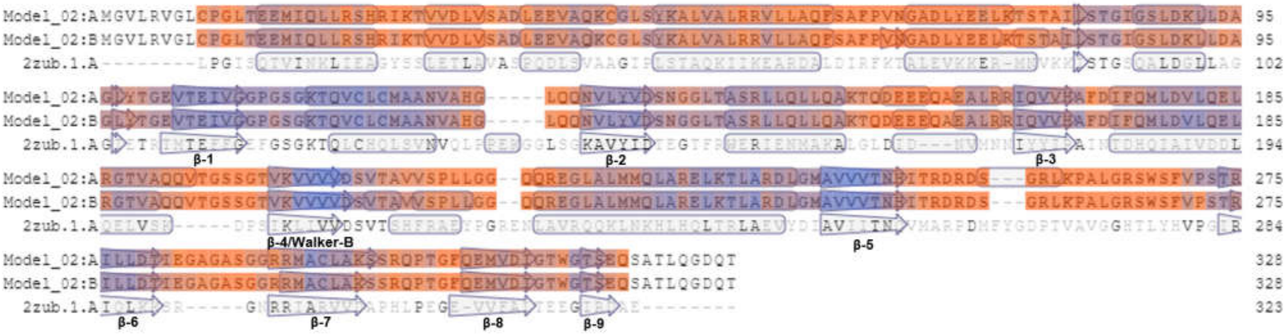

Figure S7. Mapping of 9 RAD51D C-terminal β-strands. We used the SWISS-Model server to produce a homology-modelling of Homo Sapiens RA51D (UniProt ID O75771) using as template SWISS-MODEL template library (SMTL) ID 2zub.1 (archaeal DNA repair and recombination protein RadA X-ray structure, Chang et al. [64]). The quaternary structure of the model is predicted as and homo-dimer The figure shows the model (homodimers A and B)-template alignment. Secondary structure has been annotated with default DSSP (9 predicted RAD51D C-terminal β–strands are annotated as arrows).

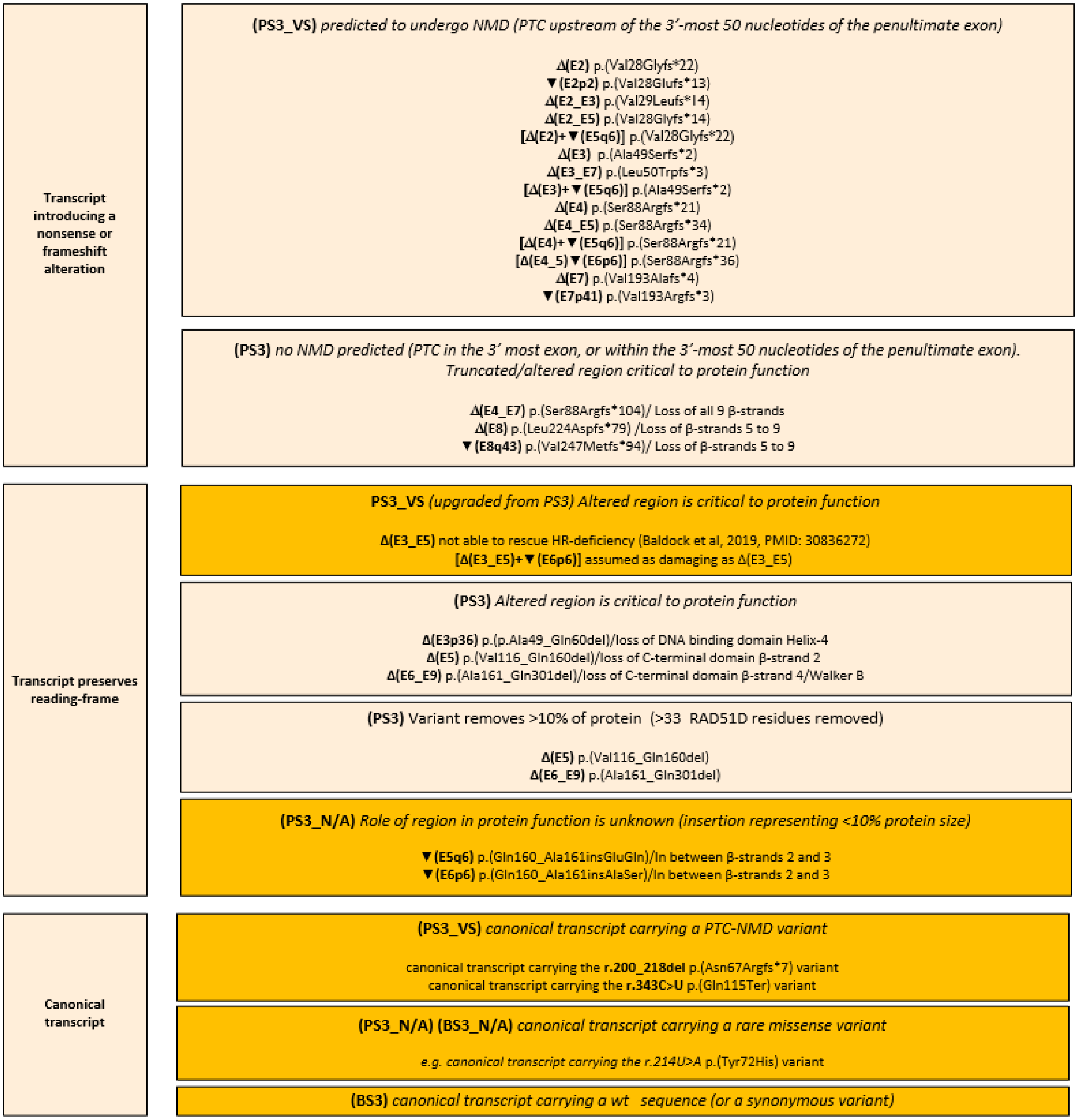

Figure S8. PS3/BS3 annotation of *RAD51D*-altered and canonical transcripts. We have adapted the PVS1 decision tree proposed by the ClinGen Sequence Variant Interpretation Working Group (ClinGen SVI) to the specific purpose of determining the strength of the loss-of-function evidence for all *RAD51D* transcripts (altered and canonical) produced by our pSAD analysis. The flowchart indicates different criteria and code strengths, as proposed by the ClinGen SVI, with three modifications (color highlighted): (i) the ClinGEN SVI do not address the eventuality of small insertions in the coding sequence. Therefore, for the sole purpose of this study, we propose a novel criteria/code strength that we think preserves the rationale of the original ClinGen SVI proposal. According to this ad-hoc criteria, small in-frame alterations in the coding sequence not targeting critical regions do not qualify for any pathogenic code strength). Note that some in-frame alterations qualify for PVS1_Strong based on size (alteration removes >10% of protein) and function (loss of region critical to protein function). (ii) Based on functional data, we propose that in-frame skipping of RAD51D exons 3, 4 and 5 is a

bona-fide loss-of-function alteration. (iii) For complex splicing read-outs that combine both altered and canonical transcripts, we have assigned also a PS3/BS3 code strength to canonical transcripts (based on coding potential).

Table S1. Mutagenesis primers for RAD51D variants and microdeletions.

Variant	Exon/Intron	Primers (5′→3′)
c.83-2A>G	ivs1	GCTATACGTGTTTTGTTTTCGGTGGTGGACCTGGTTTCT
		AGAAACCAGGTCCACCACCGAAAACAAAACACGTATAGC
c.83-4_83-3delinsAG	ivs1	CCGCTATACGTGTTTTGTTTAGAGTGGTGGACCTGGTTTCTG
		CAGAAACCAGGTCCACCACTCTAAACAAAACACGTATAGCGG
c.145-2A>G	ivs2	GCCCTTTGGCGGGCCCTGGTTGCCCTGAGGCGGGTGCT
		AGCACCCGCCTCAGGGCAACCAGGGCCCGCCAAAGGGC
c.163C>T (p.Arg55Trp)	ex3	CCCTGGTTGCCCTGAGGTGGGTGCTGCTGGCTCAGTT
		AACTGAGCCAGCAGCACCCACCTCAGGGCAACCAGGG
c.164G>A (p.Arg55Gln)	ex3	GGCCCTGGTTGCCCTGAGGCAGGTGCTGCTGGCTCAGTTC
		GAACTGAGCCAGCAGCACCTGCCTCAGGGCAACCAGGGCC
c.171G>A (p.Leu57=)	ex3	CTGGTTGCCCTGAGGCGGGTGCTACTGGCTCAGTTC
		GAACTGAGCCAGTAGCACCCGCCTCAGGGCAACCAG
c.175G>T (p.Ala59Ser)	ex3	CTGGTTGCCCTGAGGCGGGTGCTGCTGTCTCAGTTC
		GAACTGAGACAGCAGCACCCGCCTCAGGGCAACCAG
c.180G>A (p.Gln60=)	ex3	GGCGGGTGCTGCTGGCTCAATTCTCGGCTTTCCCCGTG
		CACGGGGAAAGCCGAGAATTGAGCCAGCAGCACCCGCC
c.180G>T (p.Gln60His)	ex3	GGCGGGTGCTGCTGGCTCATTTCTCGGCTTTCCCCGTG
		CACGGGGAAAGCCGAGAAATGAGCCAGCAGCACCCGCC
c.184T>A (p.Ser62Thr)	ex3	GTTGCCCTGAGGCGGGTGCTGCTGGCTCAGTTCACGGC
		GCCGTGAACTGAGCCAGCAGCACCCGCCTCAGGGCAAC
c.185C>T (p.Ser62Leu)	ex3	TGAGGCGGGTGCTGCTGGCTCAGTTCTTGGCTTTCCCC
		GGGGAAAGCCAAGAACTGAGCCAGCAGCACCCGCCTCA
c.186G>A (p.Ser62=)	ex3	TGCTGGCTCAGTTCTCAGCTTTCCCCGTGAATGGCGCT
		AGCGCCATTCACGGGGAAAGCTGAGAACTGAGCCAGCA
c.187G>A (p.Ala63Thr)	ex3	GGGTGCTGCTGGCTCAGTTCTCGACTTTCCCCGTGAAT
		ATTCACGGGGAAAGTCGAGAACTGAGCCAGCAGCACCC
c.187G>C (p.Ala63Pro)	ex3	GGTGCTGCTGGCTCAGTTCTCGCCTTTCCCCGTGAATG
		CATTCACGGGGAAAGGCGAGAACTGAGCCAGCAGCACC
c.195C>T (p.Pro65=)	ex3	GTGCTGCTGGCTCAGTTCTCGGCTTTCCCTGTGAATGG
		CCATTCACAGGGAAAGCCGAGAACTGAGCCAGCAGCAC
c.196G>A (p.Val66Met)	ex3	TGCTGCTGGCTCAGTTCTCGGCTTTCCCCATGAATGGC
		GCCATTCATGGGGAAAGCCGAGAACTGAGCCAGCAGCA
c.198G>T (p.Val66=)	ex3	GCTTTCCCCGTTAATGGCGCTGATCTCTACGAGGAACT
		AGTTCCTCGTAGAGATCAGCGCCATTAACGGGGAAAGC
c.199A>G (p.Asn67Asp)	ex3	CCCCGTGGATGGCGCTGATCTCTACGAGGAACTGAAGA
		TCTTCAGTTCCTCGTAGAGATCAGCGCCATCCACGGGG
c.200_218del (p.Asn67Argfs*3)	ex3	GCTCAGTTCTCGGCTTTCCCCGTGAGGAACTGAAGACCTCCAC-TGCCATC
		GATGGCAGTGGAGGTCTTCAGTTCCTCACGGGGAAAGCCGA-GAACTGAGC
c.202G>A (p.Gly68Ser)	ex3	CTTTCCCCGTGAATAGCGCTGATCTCTACGAGGAACTG
		CAGTTCCTCGTAGAGATCAGCGCTATTCACGGGGAAAG
c.208G>A (p.Asp70Asn)	ex3	TGAATGGCGCTAATCTCTACGAGGAACTGAAGACCTCC
		GGAGGTCTTCAGTTCCTCGTAGAGATTAGCGCCATTCA
c.209A>T (p.Asp70Val)	ex3	TCCCCGTGAATGGCGCTGTTCTCTACGAGGAACTGAAG
		CTTCAGTTCCTCGTAGAGAACAGCGCCATTCACGGGGA
c.211C>T (p.Leu71Phe)	ex3	GTGAATGGCGCTGATTTCTACGAGGAACTGAAGACC
		GGTCTTCAGTTCCTCGTAGAAATCAGCGCCATTCAC
c.213C>T (p.Leu71=)	ex3	GAATGGCGCTGATCTTTACGAGGAACTGAAGACCTCCA

		TGGAGGTCTTCAGTTCCTCGTAAAGATCAGCGCCATTC
c.214T>C (p.Tyr72His)	ex3	GAATGGCGCTGATCTCCACGAGGAACTGAAGACCTCCA
		TGGAGGTCTTCAGTTCCTCGTGGAGATCAGCGCCATTC
c.216C>T (p.Tyr72=)	ex3	CGTGAATGGCGCTGATCTCTATGAGGAACTGAAGACCT
		AGGTCTTCAGTTCCTCATAGAGATCAGCGCCATTCACG
c.217G>A (p.Glu73Lys)	ex3	TGGCGCTGATCTCTACAAGGAACTGAAGACCTCCACTG
		CAGTGGAGGTCTTCAGTTCCTTGTAGAGATCAGCGCCA
c.224T>C (p.Leu75Pro)	ex3	TACGAGGAACCGAAGACCTCCACTGCCATCCTGTCCAC
		GTGGACAGGATGGCAGTGGAGGTCTTCGGTTCCTCGTA
c.234C>T (p.Ser78=)	ex3	CGAGGAACTGAAGACCTCTACTGCCATCCTGTCCACTG
		CAGTGGACAGGATGGCAGTAGAGGTCTTCAGTTCCTCG
c.243C>T (p.Ile81=)	ex3	CGAGGAACTGAAGACCTCCACTGCCATTCTGTCCACTG
		CAGTGGACAGAATGGCAGTGGAGGTCTTCAGTTCCTCG
c.263+6T>C	ivs3	CTGGCATTGGCAGGTTTGCGTGTGTACTTGGGGAAGAA
		TTCTTCCCCAAGTACACACGCAAACCTGCCAATGCCAG
c.343C>T (p.Gln115Ter)	ex4	CCCAGGTAGCGGCAAAACTTAGGTACATGTGAGGCCAGC
		GCTGGCCTCACATGTACCTAAGTTTTGCCGCTACCTGGG
c.345+2T>C	ivs4	GGTAGCGGCAAAACTCAGGCACATGTGAGGCCAGCAGTC
		GACTGCTGGCCTCACATGTGCCTGAGTTTTGCCGCTACC
c.480+1G>A	ivs5	CAGGATGAGGAGGAACAGATAAGGGCAGGATGCTGGGTT
		AACCCAGCATCCTGCCCTTATCTGTTCCTCCTCATCCTG
c.476_480+1dup	ivs5	CCAGGATGAGGAGGAACAGGAACAGGTAAGGGCAG-GATGCTGGGTT
		AACCCAGCATCCTGCCCTTACCTGTTCCTGTTCCTCCTCATCCTGG
c.481-8C>A	ivs5	CAAAGTCCTTGCTTTTCTTTTCCACAGCTTCAGGCAGAA
		TTCTGCCTGAAGCTGTGGAAAAGAAAAGCAAGGACTTTG
c.577-2A>G	ivs6	GCTTCCCCCACATTCCTTCGGGTGACTGGTTCTTCAGG
		CCTGAAGAACCAGTCACCCGAAGGAATGTGGGGGAAGC
c.738+1G>A	ivs8	CTTGGCATGGCAGTGGTGATGAGGAAGCAGGCTTGGC
		GCCAAGCCTGCTTCCTCATCACCACTGCCATGCCAAG
c.738+1G>A	ivs8	CTTGGCATGGCAGTGGTGATGAGGAAGCAGGCTTGGC
		GCCAAGCCTGCTTCCTCATCACCACTGCCATGCCAAG
**Artificial variants**		
c.163C>G (p.Arg55Gly)	ex3	CCCTGGTTGCCCTGAGGGGGGTGCTGCTGGCTCAGTT
		AACTGAGCCAGCAGCACCCCCCTCAGGGCAACCAGGG
c.178C>T (p.Gln60Ter)	ex3	GTGCTGCTGGCTTAGTTCTCGGCTTTCCCCGTGAATG
		CATTCACGGGGAAAGCCGAGAACTAAGCCAGCAGCAC
c.212T>V (T>A, p.Leu71His) (T>C, p.Leu71Pro) (T>G, p.Leu71Arg)	ex3	GAATGGCGCTGATCVCTACGAGGAACTGAAGACCTCCA
		TGGAGGTCTTCAGTTCCTCGTAGBGATCAGCGCCATTC
c.213C>R (C>A, p. Leu71=) (C>G, p. Leu71=)	ex3	GAATGGCGCTGATCTRTACGAGGAACTGAAGACCTCCA
		TGGAGGTCTTCAGTTCCTCGTAYAGATCAGCGCCATTC
c.214T>R (T>A, p.Tyr72Asn) (T>G, p.Tyr72Asp)	ex3	GAATGGCGCTGATCTCRACGAGGAACTGAAGACCTCCA
		TGGAGGTCTTCAGTTCCTCGTYGAGATCAGCGCCATTC
c.215A>B (A>C, p.Tyr72Ser)	ex3	GAATGGCGCTGATCTCTBCGAGGAACTGAAGACCTCCA

(A>G, p.Tyr72Cys) (A>T, p.Tyr72Phe)		
		TGGAGGTCTTCAGTTCCTCGVAGAGATCAGCGCCATTC
c.216C>R (C>A, p.Tyr72Ter) (C>G, p.Tyr72Ter)	ex3	GAATGGCGCTGATCTCTARGAGGAACTGAAGACCTCCA
		TGGAGGTCTTCAGTTCCTCYTAGAGATCAGCGCCATTC
c.217G>Y (G>C, p.Glu73Gln) (G>T, p.Glu73Ter)	ex3	GAATGGCGCTGATCTCTACYAGGAACTGAAGACCTCCA
		TGGAGGTCTTCAGTTCCTRGTAGAGATCAGCGCCATTC
**Exon 3 Microdeletions**		
R51D_c.147del30	ex3	AGCAGTCTGGGGCCCTTTGGCAGGCTCAG-TTCTCGGCTTTCCCCGTGAAT
		ATTCACGGGGAAAGCCGA-GAACTGAGCCTGCCAAAGGGCCCCAGACTGCT
R51D_c.172del35	ex3	CCTGGTTGCCCTGAGGCGGGTGCTGTGATCTCTACGAG-GAACTGAAGACC
		GGTCTTCAGTTCCTCGTAGAGATCACAGCACCCGCCTCAGGG-CAACCAGG
R51D_c.202del34	ex3	TCAGTTCTCGGCTTTCCCCGTGAATCTGCCATCCTGTCCACTGG-CATTGG
		CCAATGCCAGTGGACAGGATGGCAGATTCACGGGGAAAGCCGA-GAACTGA
R51D_c.231del30	ex3	CTGATCTCTACGAGGAACTGAAGACCAGGTTTGTGTGTGTACTT-GGGGAA
		TTCCCCAAGTACACACACAAACCTGGTCTTCAGTTCCTCGTAGA-GATCAG
R51D-c.177del11	ex3	TTGCCCTGAGGCGGGTGCTGCTGGCCTTTCCCCGTGAATGGCGCTG ATCT
		AGATCAGCGCCATTCACGGGGAAAGGCCAGCAG-CACCCGCCTCAGGGCAA
R51D-c.186del10	ex3	GGCGGGTGCTGCTGGCTCAGTTCTCGTGAATGGCGCTGATCTC-TACGAGG
		CCTCGTAGAGATCAGCGCCATTCACGAGAACTGAGCCAGCAG-CACCCGCC
R51D-c.194del10-fw	ex3	CTGCTGGCTCAGTTCTCGGCTTTCCCGCTGATCTCTACGAG-GAACTGAAG
		CTTCAGTTCCTCGTAGAGATCAGCGGGAAAGCCGA-GAACTGAGCCAGCAG
R51D-c.202del10-fw	ex3	TCAGTTCTCGGCTTTCCCCGTGAATTCTACGAGGAACTGAAGAC-CTCCAC
		GTGGAGGTCTTCAGTTCCTCGTAGAATTCACGGGGAAAGCCGA-GAACTGA
R51D-c.210del10-fw	ex3	CGGCTTTCCCCGTGAATGGCGCTGAGAACTGAAGACCTCCAC-TGCCATCC
		GGATGGCAGTGGAGGTCTTCAGTTCTCAGCGCCATTCACGGG-GAAAGCCG
R51D-c.218del10-fw	ex3	CCCGTGAATGGCGCTGATCTCTACGGACCTCCAC-TGCCATCCTGTCCACT
		AGTGGACAGGATGGCAGTGGAGGTCCGTAGA-GATCAGCGCCATTCACGGG
R51D-c.226del10-fw	ex3	TGGCGCTGATCTCTACGAGGAACTGCTGCCATCCTGTCCACTGG-CATTGG

		CCAATGCCAGTGGACAGGATGGCAGCAGTTCCTCGTAGA-GATCAGCGCCA
R51D-c.234del10	ex3	ATCTCTACGAGGAACTGAAGACCTCCTGTCCACTGGCATTGG-CAGGTTTG
		CAAACCTGCCAATGCCAGTGGACAGGAGGTCTTCAGTTCCTCG-TAGAGAT

Table S2. Bioinformatics analysis of *RAD51D* variants with Max Ent Score [16,24,25].

RAD51D Variants [1]	Exon/Intron	MES wt	MES mut	MES Score Change [2]	MES De Novo SS-wt [3]	MES De Novo SS-mut [3]	MES Score Change [2]
c.83T>C	Ex2	8.52	9.09	+6.69			
c.83-7T>G	IVS1/Ex2	8.52	7.54	−11.5			
c.83-6T>C	IVS1/Ex2	8.52	7.83	−8.1			
c.83-5T>G	IVS1/Ex2	8.52	5.35	−37.21			
c.83-4T>C	IVS1/Ex2	8.52	8.08	−5.16			
c.83-4_83-3delinsAG	IVS1/Ex2	8.52	8.08	-		5.42<br>2 nt upstream	
c.83-2A>G	IVS1/Ex2	8.52	0.56	−93.43	−5.18	3.56	+168.73<br>1 nt upstream
c.145-10C>T	IVS2-/Ex3	2.43<br>NNSplice 0.66	3.02<br>0.71	-			
c.145-4G>A	IVS2-/Ex3	2.43<br>NNSplice 0.66	3.18<br>0.79	+230.86			
c.145-4G>T	IVS2-/Ex3	2.43<br>NNSplice 0.66	1.61<br>0.80	-			
c.145-4_145-3del-GCinsTT	IVS2-/Ex3	2.43<br>NNSplice 0.66	1.45<br>0.82	-			
c.145-2A>G	IVS2-/Ex3	2.43<br>NNSplice 0.66	−5.52<br><0.4	-			
c.146C>T	Ex3	2.43<br>NNSplice 0.66	3.66<br>0.78	-			
c.263+6T>C	Ex3/IVS3	7.44	4.86	−34.68			
c.263+7G>A	Ex3/IVS3	7.44	-	-			
c.264-8G>A	IVS3/Ex4	7.35	7.79	+3.27			
c.264-8G>C	IVS3/Ex4	7.35	9.04	+22.99			
c.343C>T	Ex4	7.79	4.36	−44.03			
c.345+2T>C	Ex4/IVS4	7.79	0.04	−99.49			
c.345+5A>G	Ex4/IVS4	7.79	10.65	+36.71			
c.346-9delC	IVS4/Ex5	9.18	6.21	−32.35			
c.346-7C>A	IVS4/Ex5	9.18	7.04	−23.31			
c.347T>C	Ex5	9.18	8.32	−9.37	4.05	4.61	+13.83<br>20 nt<br>downstream
c.478C>G	Ex5	11.08	10.28	−7.22			
c.479A>G	Ex5	11.08	10.17	−8.21			
c.480+1G>A	Ex5/IVS5	11.08	2.9	−73.83			
c.480+1_480+2in-sAACAGG	Ex5/IVS5	11.08	0.5	−95.49			
c.480+6G>A	Ex5/IVS5	11.08	10.77	−2.8			
c.480+9A>G	Ex5/IVS5	11.08	-	-			
c.481-8C>A	IVS6/Ex6	8.21	1.75	−78.68	3.02	11.06	+266.23<br>7 nt upstream
c.481-8C>T	IVS6/Ex6	8.21	8.54	+4.02			
c.481-7G>A	IVS6/Ex6	8.21	8.53	+3.9			

c.481-5T>C	IVS6/Ex6	8.21	7.57	−7.8			
c.575A>G	Ex6	9.6	8.19	−14.69			
c.577-6C>A	IVS6/Ex7	10.36	8.67	−16.31			
c.577-2A>G	Ex7	10.36	2.41	−76.74	3.92	4.13	+5.36 17 nt downstream
c.666A>G	Ex7	10.47	9.25	−11.65			
c.667+9T>C	Ex7/IVS7	10.47	-	-			
c.668-9C>T	IVS7/ex8	10.99	11.31	+2.91			
c.668-7_-5dup	IVS7/ex8	10.99	11.77	+7.1			
c.668-4G>A	IVS7/ex8	10.99	10.58	−3.73			
c.738+1G>A	Ex8/IVS8	6.13	−2.05	−133.44			
c.738+3G>A	Ex8/IVS8	6.13	8.37	+36.54			
c.739-7G>T	IVS8/Ex9	9.9	11.23	+13.43			
c.739-4G>T	IVS8/Ex9	9.9	9.81	−0.91			
c.739-3C>T	IVS8/Ex9	9.9	9.45	−4.55			
c.740T>C	Ex9	9.9	8.62	−12.93			

[1] Selected variants are in red. Potential spliceogenic variants were selected according to the following criteria: (i) MES score changes (>15%) except variants of the polypyrimidine (Pyr) tract; (ii) creation of alternative sites. Given that Pyr-tract variants use to have weak impacts on splicing (1–3), we selected only those that concurrently fitted criteria (i) and (ii). [2] MES score changes (Δ%), wild type (wt) vs. mutant (mut). [3] De novo: predicted creation of new alternative splice sites. Exon 3 variants (34): c.153T>C, c.160A>C, c.161G>T, c.163C>T, c.164G>A, c.166delG, c.171G>A, c.175G>T, c.180G>A, c.180G>T, c.184T>A, c.185C>T, c.186G>A, c.187G>C, c.187G>A, c.195C>T, c.196G>A, c.198G>T, c.199A>G, c.200_218del, c.202G>A, c.208G>A, c.209A>T, c.211C>T, c.213C>T, c.214T>C, c.216C>T, c.217G>A, c.224T>C, c.234C>T, c.243C>T, c.252T>A, c.258T>C and c.260G>A.

Table S3. RNA and protein HGVS annotations according to transcript ENST00000345365.10.

Transcript [1]	RNA-HGVS	Protein-HGVS
Splice-site variants		
Δ(E2)	r.83_144del	p.Val28Glyfs*22
▼(E2p2)	r.83-1_83ins(83-2_83-1)	p.Val28Serfs*43
Δ(E2_3)	r.83_263del	p.Val29Leufs*14
Δ(E2_5)	r.83_480del	p.Val28Glyfs*14
[Δ(E2)▼(E5q6)]	r.[83_144del; 480_480+1ins(480+1_480+6)]	p.Val28Glyfs*22
Δ(E3)	r.145_263del	p.Ala49Serfs*2
Δ(E3_5)	r.145_480del	p.Ala49_Gln160del
Δ(E3_7)	r.145_667del	p.Leu50Trpfs*4
[Δ(E3)▼(E5q6)]	r.[145_263del; 480_480+1ins(480+1_480+6)]	p.Ala49Serfs*2
[Δ(E3_5)▼(E6p6)]	r.[145_480del; 481-1_481ins(481-6_481-1)]	p.[Ala50_Gln160del; Gln160_Ala161Ins(LeuGln)]
Δ(E4)	r.264_345del	p.Ser88Argfs*21
Δ(E4_5)	r.264_480del	p.Ser88Argfs*34
Δ(E4_7)	r.264_667del	p.Ser88Argfs*104
[Δ(E4)▼(E5q6)]	r.[264_345del; 480_480+1ins(480+1_480+6)]	p.Ser88Argfs*21
[Δ(E4_5)▼(E6p6)]	r.[264_480del; 481-1_481ins(481-6_481-1)]	p.Ser88Argfs*36
Δ(E5)	r.346_480del	p.Val116_Gln160del
▼(E5q6)	r.480_480+1ins(480+1_480+6)	p.160Gln_161AlaIns(ValArg)
Δ(E6_9)	r.481_903del	p.Ala161_Gln301del
▼(E6p6)	r.481-1_481ins(481-6_481-1)	p.Gln160_Ala161Ins(LeuGln)
Δ(E7)	r.577_667del	p.Val193Alafs*4
▼(E7p41)	r.577-1_577ins(577-41_577-1)	p.Val193Argfs*6
Δ(E8)	r.668_738del	p.Gly224Aspfs*79
▼(E8q43)	r.[738_739ins(738+1_738+43)]	p.Val247Metfs*94
SRE variants		
Δ(E3p36) (exonic)	r.145_180del	p.Ala49_Gln60del

[1] Transcripts were described with a combination of the following symbols: ▼ (incorporation of intronic sequences that are not present in the reference transcript), Δ (skipping of exonic sequences that are present in the reference transcript), E (exon), p (new acceptor site), q (new donor site) and a number representing the exact number of nucleotides incorporated or skipped For example, transcript ▼(E2p2) denotes the use of an alternative acceptor site 2 nucleotides upstream of exon 2, causing the addition of 2-nt to the mature mRNA.

Table S4. Functional mapping of ESEs by exonic microdeletions.

Microdeletion	Full-length Transcript	PTC-transcripts	In-frame transcripts
Wild type	73.1% ± 5.6	Δ(E4_5) (9.4% ± 3)	Δ(E3_5) (17.5% ± 5.2)
c.147_176del	55.5% ± 1.0	Δ(E3) (21.3% ± 0.2)<br>Δ(E4_5) (4.5% ± 0.2)	Δ(E3_5) (18.4% ± 0.7)
c.172_206del	47.4% ± 0.5	Δ(E3) (29.7% ± 0.5)<br>Δ(E4_5) (2.3% ± 0.8)	Δ(E3_5) (20.7% ± 0.8)
c.202_235del	-	Δ(E3) (77.3% ± 1.7)<br>Δ(E4_5) (5.8% ± 0.3)	Δ(E3_5) (16.9% ± 1.5)
c.194_203del	55.5% ± 0.4	Δ(E3) (19.7% ± 0.4)	Δ(E3_5) (24.8% ± 0.8)
c.202_211del	54.3% ± 0.6	Δ(E3) (26.7% ±0.3)	Δ(E3_5) (19.0% ± 0.7)
c.210_219del	-	Δ(E3) (75.1% ± 0.8)	Δ(E3_5) (24.9% ± 0.8)
c.218_227del	36.3% ± 1.4	Δ(E3) (35.0% ± 0.6)	Δ(E3_5) (28.7% ± 0.8)
c.226_235del	50.7% ± 0.7	Δ(E3) (21.5% ± 0.1)	Δ(E3_5) (27.8% ± 0.6)
c.231_260del	35.8% ± 2.2	Δ(E3) (8.8% ± 0.2)<br>Δ(E4_5) (9.0% ± 1.6)	Δ(E3_5) (46.4% ± 2.9)

Table S5. Accuracy of bioinformatics predictions.

Program	Selected Variants	Spliceogenic [1]	Negative	Spliceogenic [1]
HSF summary	11	8	28	19
ΔHZ_{EI}	28	20	11	7
Hot-Skip	10	8	29	19
ΔtESRseq	14	10	25	17

[1] Variants with very weak effects (<10% reduction of the canonical transcript (range = 65.8–73.1%) were not considered spliceogenic.

Table S6. Sensitivity and specificity of the splicing programs.

Program	Spliceogenic [1] (Sensitivity)		No Impact (Specificity) [2]		Accuracy [3]
	True Positive [1]	False Negative	True Negative [2]	False Positive	
**HSF**	8<br>(29.6%)	19	9/12<br>(75.0%)	3	17/39<br>(43.6%)
**ΔHZ_{EI}**	20<br>(74.1%)	7	4/12<br>(33.3%)	8	24/39<br>(61.5%)
**Hot-Skip**	8<br>(29.6%)	19	10/12<br>(83.3%)	2	18/39<br>(46.2%)
**ΔtESRseq**	10<br>(37.0%)	17	8/12<br>(66.7%)	4	18/39<br>(46.2%)

[1] Variants with very weak effects (<10% reduction of the canonical transcript (range = 65.8–73.1%) were not considered spliceogenic. Sensitivity (between parentheses) = True positive/Total number of spliceogenic variants. [2] Specificity (between parentheses) = True negative/Total number of non-spliceogenic variants. [3] Accuracy (between parentheses) was measured as [True positive + True Negative]/Total Variants.

Supplementary Methods: ACMG/AMP-like classification of 37 *RAD51D* variants based on PS3/BS3 functional evidence.

1. Predictive Codes PVS1/PP3/BP4

In our opinion, once functional splicing data is available (either from minigene analysis or patient derived RNA), predictive splicing codes should not contribute to variant classification, but being replaced by functional splicing codes. Otherwise, internal inconsistences arise in the ACMG/AMP classification system (e.g., a rare +1, +2 variant causing a PTC-NMD splicing alteration will end up us pathogenic (PVS1+PS3+PM2), while a rare nonsense variant introducing a PTC-NMD at the same location (PVS1+PM2) will end up as likely pathogenic). The issue has been extensively addressed recently [16]. Further, the ACMG/AMP system implicitly assumes that each piece of evidence contributing to the final classification is independent [65], an assumption hardly met by predictive and functional splicing codes, as most splicing analyses (including the mgR51D_ex2-9 assays reported in the present study) are performed in bioinformatically pre-selected variants. The issue has been extensively discussed elsewhere [16].

Said that, we acknowledge a role for predictive code PVS1 in the classification of *RAD51D* nonsense, and Indels variants that are spliceogenic, but express a non-negligible proportion of canonical transcripts as well (*RAD51D* c.200_218del and c.343C>T)

2. Functional Codes PS3/BS3

Several ClinGen-SVI and ClinGen expert panel documents acknowledge the use of minigenes to provide functional splicing (PS3/BS3) codes [29,31,32]. Yet, assigning a specific PS3/BS3 code strength to minigene readouts (or to RT-PCR assays performed in carriers) is far from trivial, especially if the spliceogenic variant is demonstrated to produce two or more different mRNA transcripts. High levels of naturally occurring alternative splicing, as observed for *RAD51D* [33], further complicate code strength assignment. As far as we know, neither the ClinGen-SVI nor gene-specific expert panels have addressed this issue. Here we propose deconvoluting complex read-outs into individual transcripts, assigning PS3/BS3 code strengths to each individual transcript, and latter produce a combined PS3/BS3 code strength based on expert judgment (see Section 2.3.).

PS3 code strengths were assigned to individual mRNAs following the same rationale proposed by the ClinGen-SVI for presumed loss-of-function variants (PVS1 decision tree) [30]. With this aim, we first identify biologically relevant *RAD51D* transcript(s), and RAD51D coding regions critical to protein function.

2.1. Biological Relevant RAD51D Transcript(s)

Naturally occurring alternative splicing produces a minimum of 3 different RAD51D isoforms of 328, 216, and 348 residues (sometimes referred as isoforms 1, 4, and 6). Isoform 1 is coded by mRNA NM_002878.3 (harboring an exon 3 of 119nt), isoform 4 is coded by mRNA NM_133629.2 (skipping exons 3, 4, and 5), and isoform 6 is coded by NM_001142571.2 (harboring an alternative exon 3 of 179nt). Recently, it has demonstrated that isoform 1, but not isoforms 4 or 6, is able to rescue HR-deficiency observed in RAD51D knock-out cell lines [66]. Based on this, we propose that NM_002878.3 is the only biologically relevant *RAD51D* transcript to be considered for variant classification purposes (in the context of breast and ovarian cancer susceptibility). Note that we are implicitly assuming that RAD51D isoforms coded by mRNA NM_001142571.2 and NM_133629.2 cannot rescue HR functionality, an assumption that has implications for variant classification (e.g., we propose that a PTC-NMD variant located in NM_002878.3 exon 3 a is a bona-fide *RAD51D* loss-of-function variant qualifying for a strong pathogenic code PVS1, even though isoforms 4 and 6 will not be damaged).

2.2. RAD51D Coding Regions Critical to Protein Function

Expert Panel specifications of the ACMG/AMP guidelines for the analysis of germline *RAD51D* sequence variants are not yet available (clinicalgenome.org/, last accessed on 29 December 2020). Among other drawbacks, the latter implies that we lack an authoritative reference mapping the boundaries of RAD51D regions critical to protein function. Yet, such mapping is a prerequisite to assign ACMG/AMP code strengths to PTC and in-frame splicing alterations (PVS1 decision tree) [30]. To cope with this, we have performed an in-house mapping of RAD51D critical regions. Based on current evidence, we propose that a minimum of 13 RAD51D coding regions (4 α-helix located in the N-terminal domain and 9 β-strands located in the C-terminal domain) are critical to RAD51D folding and/or function, and therefore relevant to classify in-frame alterations:

- The N-terminal domain (residues 1–83) is essential for RAD51D binding to ssDNA and XRCC2 (ternary complex), suggesting a critical role during homologous recombination. NMR spectroscopy of the human N-terminal RAD51D domain has identified four α-helix critical to proper protein folding and DNA binding. These α-helices span residues p.Glu14_Ser22 (Helix-1), p.Val28_Leu31 (Helix-2), p.Leu33_Cys43 (Helix-3), and p.Tyr47_Phe61 (Helix-4) [61]. Based on that, we decided that a strong pathogenic code (PS3) should be assigned to mRNAs with in-frame alterations deleting any of these four α-helix (Figure S3).
- Like RAD51 and other RAD51 paralogues, RAD51D features a highly conserved C-terminal ATPase domain, including canonical Walker A and B motifs for ATP hydrolysis. Yet, the role of RAD51D ATPase activity for DNA repair, if any, is controversial [66–68]. Based on that, we think that no pathogenic code strength should be applied to mRNAs with an in-frame alteration deleting the Walker A motif without deleting flanking β-strands 1 and 2 (said that we have not identify any mRNA harboring such in-frame alteration). By contrast, regardless of ATPase role, we think that a pathogenic strong code (PS3) should be assigned to mRNAs with in-frame alterations deleting the Walker-B motif that overlaps with β-strand 4 and has therefore a structural role.
- Modeling of human RAD51 and some RAD51 paralogues (RAD51B, RAD51C, XRCC2, and XRCC3) has revealed a series of 9 β–strands that form an internal β-sheet within the C-terminus domain of these proteins. Experimental data suggests that this β–sheet is critical to maintain a proper C-terminal folding. Further, since the order of the β–strands in space is different than the order in sequence [63], it is anticipated that deleting any of these β–strands will cause protein misfolding [62]. Based on that, we have assigned a pathogenic strong code (PS3) code to mRNAs with in-frame alterations deleting any of the 9 β–strands. Indeed, it can be argued that a very strong code strength (PS3_VS) is more appropriate for these mRNAs since the C-terminal domain is predicted to collapse. Yet, as far as we know there is no convincing clinical data supporting that in-frame deletions of one RAD51D C-terminal β–strands are risk associated. Lacking this evidence, we have decided to be conservative.

Since no RAD51D C-terminal protein structure exists, we have mapped 9 RAD51D C-terminal β–strands using two complementary approaches: (i) alignment of *P.furiosus* RAD50, *Mus_musculus* RAD51D, *Homo_sapiens* RAD51D and *Homo_sapiens* RAD51, followed by manual annotation of human RAD51D C-terminal β–strands based on *Mus_musculus* RAD51D annotations [62] (Figure S5), and (ii) generation of a SWISS-Model for human RA51D (Figure S6). Ultimately, the first approach is based on the X-ray structure of *Pf*RadA (Rad51) [63], while the second is based on the X-ray structure of *Sso*RadA (Rad51) [64]. Yet, both approaches produced essentially identical results, mapping human RAD51D C-terminal β-strands as follows: β-strand 1(p.101-107), β-strand 2 (p.131-136), β-strand 3 (p.166-170), β-strand 4/Walker B (p.200-207), β-strand 5 (p.244-250), β-strand 6 (p.274-280), β-strand 7 (p.291-297), β-strand 8 (p.306-311) and β-strand 9 (p.316-318).

2.3. Assigning PS3/BS3 Code Strengths to Individual Minigene Produced mRNAs

Overall, minigene-based analysis of 38 RAD51D variants have produced up to 26 different transcripts, including 14 disrupting reading-frame and predicted to undergo NMD (PTC-NMD transcripts), three disrupting reading-frame but not predicted to undergo NMD (PTC transcripts), seven preserving reading-frame (in-frame transcripts), and two uncharacterized transcripts of 487 and 1363 nucleotides (see Tables 1 and 3). In addition, full-length transcripts (either WT or carrying various exon 3 variants under investigation) were also detected. To assign PS3/BS3 code strengths to individual transcripts, we adapted the PVS1 decision tree developed by the ClinGen SVI (Figure S7):

- All 14 PTC-NMD transcripts were considered loss-of-function transcripts, and therefore assigned a pathogenic very strong (PS3_VS) code (Figure S7).
- All three PTC transcripts are predicted to eliminate regions critical to RAD51D function (a minimum of five C-terminal β-strands), and therefore assigned a pathogenic strong (PS3) code (Figure S7).
- Five in-frame transcripts, namely Δ(E3p36), Δ(E3_E5), [Δ(E3_E5) + ▼(E6p6)], Δ(E5), and Δ(E6_E9), are predicted to eliminate regions critical to RAD51D function (N-terminal DNA binding domain Helix-4, or C-terminal β-strands, or both), and therefore assigned a pathogenic strong (PS3) code. Further, regardless of the exact boundaries of critical functional regions, the latter four transcripts can be assigned a pathogenic strong (PS3) code based on the alternative rationale that these mRNAs are predicted to remove >10% of the coding region. Since RAD51D isoform 4 is not able to rescue HR-deficiency [66], mRNAs skipping exons 3, 4, and 5 are predicted to be loss-of-function, and therefore upgraded to pathogenic very strong (PS3_VS) code (Figure S7).
- Two other in-frame transcripts, ▼(E5q6) and ▼(E6p6), are predicted to introduce two residues in between β-strands 2 and 3, a coding region that we have not documented and/or predicted to be functionally relevant. Based on that, we think that these mRNAs do not provide any evidence in favor of pathogenicity and, therefore, no pathogenic code strength has been assigned (PS3_N/A) (Figure S7). While pathogenic code strengths for in-frame insertions are not directly addressed by the ClinGen-SVI PVS1 decision tree [30], we think that our proposal of PS3_N/A for in-frame insertion not targeting known critical regions, and representing <10% of the protein size, follows the same rationale.
- The uncharacterized transcripts of 487 and 1363 nucleotides were considered not to provide any evidence in favor (PS3_N/A), or against (BS3_N/A), pathogenicity, even though large size differences if compared with canonical transcripts (~356nt shorter the former, ~480 nucleotides longer the latter) predict a major impact on the coding sequence. At any rate, the 487 and 1363 nucleotide transcripts represent ≤10% of the overall expression (readouts of c.83-2A>G and c.738+1G>A assays, respectively), and are therefore disregarded for PS3/BS3 code strength assignment (see Section 2.4.).
- Canonical transcripts not harboring genetic variant (WT canonical transcripts) were assigned a strong benign (BS3) code. The same was true for canonical transcripts harboring a synonymous variant (note that we are not supporting that synonymous variants are benign, but rather that the appropriate pathogenic code strength of a synonymous variant, if any, will depend on its spliceogenic effect). For canonical transcripts carrying PTC-NMD variants, a pathogenic very strong (PS3_VS) code was assigned. Finally, for canonical transcripts carrying missense variants, neither benign nor pathogenic codes were assigned. As previously recommended by ClinGen expert panels, we have decided not to use protein-based computational prediction models for missense variants [32].

2.4. Assigning an Overall PS3/BS3 Code Strengths to Minigene Readouts

For variants producing only one transcript (or different transcripts for which we have assigned PS3/BS3 codes of equal strength), assigning a PS3/BS3 code strength to minigene readouts was straightforward. To associate a specific PS3/BS3 code strength to complex minigene read-outs (two or more transcripts with various predicted impacts on the coding sequence), we have developed the following algorithm: (i) determining the contribution of each PS3/BS3 code strength (one mRNA, or various mRNAs with the same assigned code strength) to the overall expression, (ii) disregarding PS3/BS3 code strengths representing <10% of the overall expression, (iii) if only pathogenic code strengths (PS3_VS, PS3) contributed ≥10% to the overall expression, we selected the most conservative pathogenic code strength (PS3) as an overall PS3 code strength, (iv) if both pathogenic and benign codes contribute ≥10% to the overall expression, neither pathogenic nor benign codes were assigned (i.e. the splicing assay was considered not providing any evidence in favor, or against, pathogenicity), (v) the only exception to the latter were splicing outputs in which the benign BS3 code (wt canonical transcript) represented ≥67% of the overall expression. In these cases, an overall benign code (BS3) was assigned. This exception was incorporated into the system to reflect the high level of naturally occurring alternative splicing existing at the *RAD51D* locus [33], and rightly reproduced by the p.SAD mgR51D ex2–9 minigene system. Indeed, the selected ≥67% cut-off is based on read-outs observed in the wt minigene (BS3 representing 73% ± 6% of the overall expression, PS3_VS representing the remaining ~30%, see Tables 1 and 3).

3. Pathogenic Code PM2 (Rarity Evidence)

The original ACMG/AMP guidelines defined this rarity code as absent from controls (ExAC, ESP, and/or 1000 genomes project). However, the availability of even larger control datasets (e.g., gnomAD) challenges the view that pathogenic alleles cannot be found in these datasets. Here we follow the rule proposed by the ClinGen CDH1 variant curation expert panel of ≤1 in 100,000 alleles [32]. For allele counting, we have interrogated gnomADv2.1 (global). For *RAD51D* variants with no counts in gnomADv2, we used as a proxy for allele counts data on the closest available SNP (in all cases, ≤7nt apart from the variant of interest, see Table 3).

For c.343C>T, we observed quite different allele counts for nearby rs786202507 (4nt upstream) and rs878854562 (7nt downstream) SNPs (31,370 allele counts the former and 251,220 the latter), so that we decided to use as a proxy for allele count an average (141,295 alleles).

4. Pathogenic Code PM3 (In Trans with a Pathogenic Variant in a Recessive Disorder)

Mounting evidence indicates a common genetic basis for dominant breast and ovarian cancer susceptibility and the recessive Fanconi Anemia syndrome. Yet, as far know, there is no evidence that bi-allelic pathogenic variants in *RAD51D* cause Fanconi Anemia (ClinGen curated RAD51D at https://search.clinicalgenome.org/kb/gene/HGN:9823, last accessed on 17 December 2020), so that we discarded PM3 as a possible contributor to the clinical classification of *RAD51D* variants.

5. Benign Code BS1 (Frequency Greater than Expected for Disorder)

We have calculated the maximum credible population allele frequency [69] for a *RAD51D* variant using the online application at http://cardiodb.org/allelefrequencyapp/ (accessed on 1 April 2021). As a proxy for breast and ovarian cancer prevalence in the population, we have used estimated lifetime risk of breast and ovarian cancer for UK women born after 1960 (www.cancerresearchuk.org/health-professional/cancer-statistics/risk/lifetime-risk; accessed on 1 April 2021). For breast and ovarian cancer penetrance in *RAD51D* carriers, we have used recently published estimates [3]. Allelic heterogeneity

was set at 0.1 (the variant account for 10% of all the pathogenic RAD51D alleles). Genetic heterogeneity was set at 0.01 (1% of all breast and ovarian cancers cases caused by a RAD51D germ-line mutation). We think that we are probably overestimating RAD51D allelic and genetic heterogeneity. Indeed, the actual proportion of breast and ovarian cancers caused by *RAD51D* germ-line pathogenic variants is probably close to 0.1% and 0.3% respectively [5,70,71], with no evidence for highly recurrent variants in the general population. Calculations were as follows:

- breast cancer prevalence at 1:7, allelic heterogeneity at 0.1, genetic heterogeneity at 0.01, penetrance at 0.2, and confidence at 0.95, to provide a maximum credible population allele frequency of 0.000357 (46 in 100,000 gnomAD alleles).
- ovarian cancer prevalence at 1:50, allelic heterogeneity at 0.1, genetic heterogeneity at 0.01, penetrance at 0.13, and confidence at 0.95 to provide a maximum credible population allele frequency of 7.69e-05 (15 in 100,000 gnomAD alleles).

Based on these analyses, we decided to select an intermediate value (30 in 100,000 gnomAD alleles) as a safe threshold for a strong benign code BS3.
